# Pre-, Intra-, and Post-Operative Evaluation of Extraocular Muscle Insertions Using Optical Coherence Tomography: A Comparison of Four Devices

**DOI:** 10.3390/jcm8101732

**Published:** 2019-10-19

**Authors:** Matthew S. Pihlblad, Andrew Troia, Sapna Tibrewal, Parth R. Shah

**Affiliations:** Department of Ophthalmology, UPMC Children’s Hospital of Pittsburgh, University of Pittsburgh, Pittsburgh, PA 15224, USA

**Keywords:** optical coherence tomography, extraocular muscles, rectus muscle insertions, strabismus surgery

## Abstract

OCT (optical coherence tomography) is widely used in ophthalmology and pediatric ophthalmology, but limited research has been done on the use of OCT in strabismus. This study investigates the use of different OCT machines to image rectus muscle insertions pre-, intra-, and post-operatively in pediatric strabismus patients. The OCT machines used in the study were a Bioptigen (Leica Microsystems Inc., Buffalo Grove, IL, USA), Spectralis HRA+OCT with Anterior Segment Module (Heidelberg Engineering, Heidelberg, Germany), Visante (Carl Zeiss, Oberkochen, Germany), and Zeiss Rescan 700 (Carl Zeiss, Oberkochen, Germany). Measurements from the machines were compared with the caliper distance measured during the strabismus surgery before disinsertion or after reattachment. The OCT machines had moderate (Bioptigen: 0.62) to good intraclass correlation coefficients (Rescan: 0.83, Spectralis: 0.85, Visante: 0.88) with intra-operative measurements. To our knowledge, this is the first study to use an operating microscope with integrated intra-operative OCT to image rectus muscle insertions. OCT is a useful tool in strabismus surgical patients in the pre-, intra-, and post-operative settings, particularly in patients who have had previous surgery, when the muscle insertion is unknown. The ability to accurately image rectus muscle insertions has significant implications for the management of strabismus patients.

## 1. Introduction

Strabismus surgery on patients with previous strabismus, orbital, or other ophthalmic surgeries can be challenging, and the response to surgery less predictable than in patients without prior ocular surgery. The need for multiple strabismus surgeries is not uncommon [1]. The subsequent surgery or surgeries can frequently be performed by a different surgeon, with few details of the original surgery that may have been performed years or decades prior. Even with the previous operative reports or a history from the patient, the location of the extraocular muscles (EOMs) can be different than expected. Slit lamp examination can give clues to the prior surgery, but not specifics. Knowing the location of the EOMs prior to surgery and what previous surgery was performed can be very beneficial to the surgeon. Several techniques have been used to image the EOM pre-, intra-, and post-operatively to help plan surgery and reduce surgical exploration and time, including computed tomography (CT), magnetic resonance imaging (MRI), B-Scan ultrasonography (US), and ultrasound biomicroscopy (UBM) [2,3,4,5,6,7,8,9,10,11]. More recent studies have used the optical coherence tomography (OCT) to image EOM insertion on un-operated-on and previously operated-on muscles, but to our knowledge, none have used an operating microscope with integrated intra-operative OCT [12,13,14,15,16,17,18,19,20,21,22,23,24,25,26,27,28,29]. 

## 2. Experimental Section

A prospective study of pediatric strabismus patients (≤18 years old at enrollment) scheduled for strabismus surgery was enrolled. All subjects or parents signed informed consent, depending on age, for inclusion before they participated in the study. The study was conducted in accordance with the Declaration of Helsinki, and the protocol was approved by the University of Pittsburgh Institutional Review Board. OCT was used to image the rectus muscle insertions on pediatric strabismus patients pre-operatively, intra-operatively, and post-operatively. The different OCT machines used were a Bioptigen, Inc. (Leica Microsystems Inc., Leica Microsystems Inc., Buffalo Grove, IL, USA), Spectralis HRA+OCT with Anterior Segment Module (Heidelberg Engineering, Heidelberg, Germany), Visante (Carl Zeiss, Oberkochen, Germany), and Zeiss Rescan 700 (Carl Zeiss, Oberkochen, Germany). The measurements were compared to the handheld caliper distance measured during the strabismus surgery prior to disinsertion and where reattached to the sclera after surgery was performed. The muscle(s) scheduled to be operated on were imaged pre-operatively if the patients were 3 years or older and cooperative for OCT imaging with the Spectralis and Visante, as well as at the post-operative 1 month and the 4–6 month visit. All patients of any age had OCT images taken intra-operatively before the start of the surgery with the Bioptigen and Rescan. 

A fixation light or fixation target was used to have patients look in different gaze positions, in order to image the muscle insertions pre-operatively and post-operatively. For example, the lateral rectus (LR) insertion was imaged with the eye in adduction. Of the images acquired, the highest quality image, as determined by the interpreting pediatric ophthalmologist (MP), was selected to measure the distance from the limbus to the muscle insertion with digital calipers incorporated into each machine’s software.

**Figure 1:**
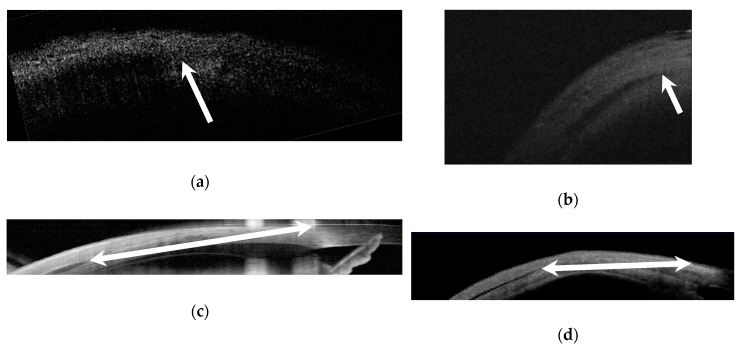
Anterior segment optical coherence tomography of a lateral rectus muscle insertion: (**a**) Bioptigen, (**b**) Rescan, (**c**) Spectralis, and (**d**) Visante. The rectus muscle insertion was located as a dark structure tapering toward the limbus between the sclera and conjunctiva/Tenon’s. Arrows mark the insertion in (**a**,**b**); the limbus was identified intra-operatively, and then measured to the insertion on the optical coherence tomography (OCT) with handheld calipers. Double arrows mark the insertion and limbus in (**c**,**d**), and calipers on the machine measured the distance.

The EOM insertion in this study was defined as the most anterior point of the muscle tendon, which is visible as a dark structure tapering toward the limbus between the sclera and conjunctiva/Tenon’s (See Figure 1). The limbus was defined on the Visante as the transition between the corneal epithelium and the conjunctival epithelium, as described by Rosetto et al. [27]. The limbus was marked on the external photo with the Spectralis. Measurements were taken using digital calipers integrated in the AS-OCT software by a pediatric ophthalmologist on the Visante and Spectralis. All images were obtained by a single, well-experienced ophthalmic photographer pre-operatively and post-operatively, and all intraoperative measurements by the operating pediatric ophthalmologist (MP). 

**Figure 2:**
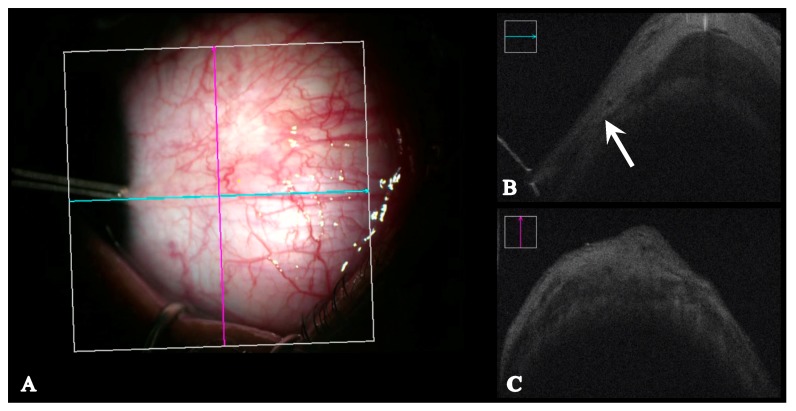
Anterior segment optical coherence tomography (integrated intraoperative microscope, Zeiss Rescan 700 (Carl Zeiss, Germany)) of a previously resected medial rectus muscle. (**A**): intraoperative view. (**B**): arrow marks muscle insertion. (**C**): cross section of muscle shown.

The Bioptigen and Rescan (Figure 2) were used to capture intra-operative measurements prior to the start of the strabismus surgery. The 10 mm anterior probe was used to capture the images with the Bioptigen, which was either hand-held or placed in an adjustable custom mount. The Rescan was used in the anterior segment mode. Intra-operatively, the eye was brought the direction opposite of the desired muscle to be imaged, with forceps grasping the episclera near the limbus. The limbus was identified clinically, and handheld calipers were used to make a shadow on the intra-operative OCT image at the anterior insertion of the tendon, or to mark the insertion on the conjunctiva and then measure to the limbus.

The “intra-operative” measurements during the surgical procedure were obtained with handheld calipers from the limbus to the most anterior insertion of the muscle centrally after “cleaning” the muscle of Tenon’s, the surrounding connective tissue, and any scar. After the muscle was reattached to the sclera during surgery, the new insertion to limbus was measured with handheld calipers. 

To compare the different measurements, Bland–Altman plots were performed, along with intraclass correlation coefficients (ICCs). The statistical analysis was done with Excel 2013 (Microsoft, Redmond, WA, USA).

## 3. Results

**Figure 3:**
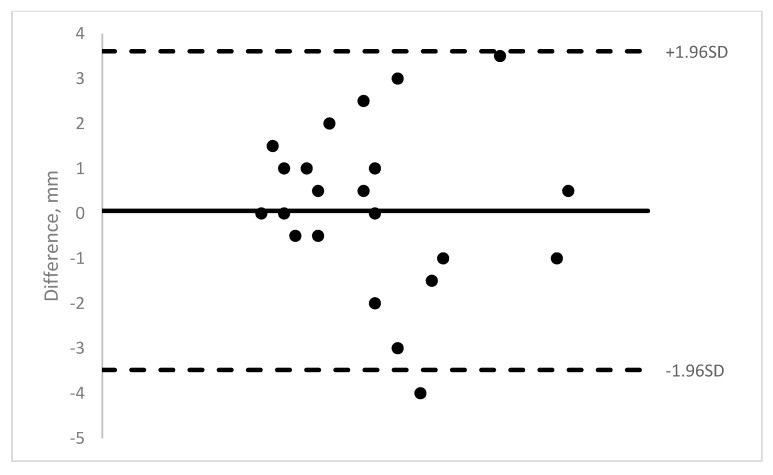
Bioptigen Bland–Altman plot of intra-operative measurements versus OCT measurement.

**Figure 4:**
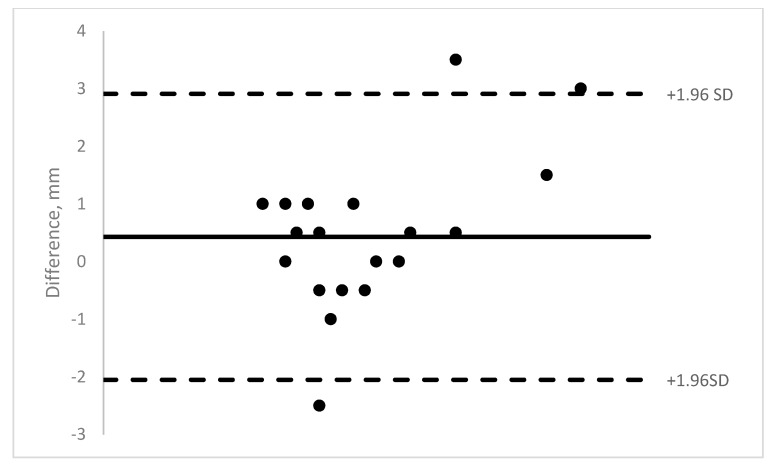
Rescan Bland–Altman plot of intra-operative measurements versus OCT measurement.

**Figure 5:**
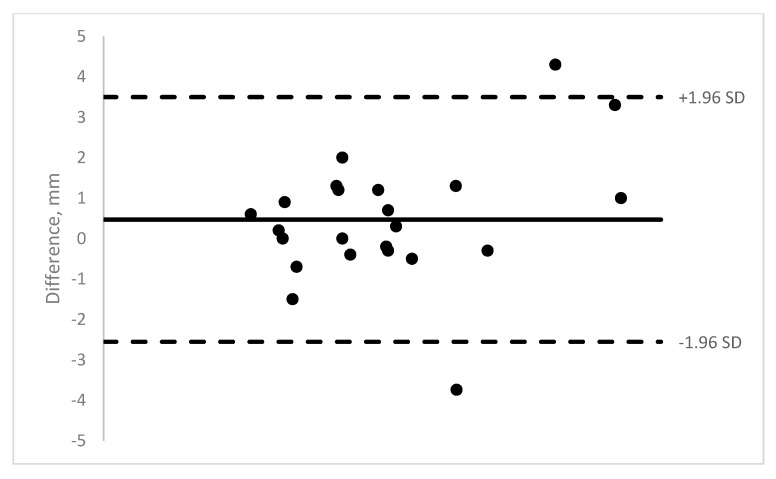
Spectralis Bland–Altman plot of intra-operative measurements versus OCT measurement.

**Figure 6:**
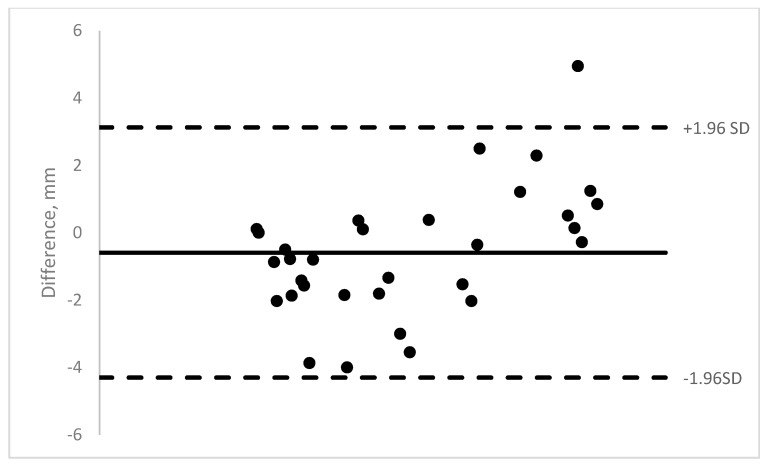
Visante Bland–Altman plot of intra-operative measurements versus OCT measurement.

Nineteen pediatric patients were enrolled in the study. Ten were males and 9 were females. The age range was 11 months to 13 years (mean = 5.8 years). A total of 140 OCT images of 40 muscles were obtained (14 medial rectus, 22 lateral rectus, and 4 inferior rectus). The majority of the rectus muscle insertions were able to be imaged (58–89% depending on machine). Those results show moderate to good ICCs (0.62–0.88) (See Table 1). See Figure 3, Figure 4, Figure 5 and Figure 6 for Bland–Altman plots for each OCT machine. The percentage of muscles measuring ≤1 mm was higher pre-operatively than post-operatively (Spectralis: 89% vs. 54%; and Visante: 45% vs. 31%). See Table 2 for the list of patients able to be imaged with all four optical coherence tomography machines.

## 4. Discussion

OCT is a useful tool in strabismus surgical patients, particularly for previously operated patients where the muscle insertion may not be known. OCT can be useful pre-operatively, intra-operatively, and post-operatively. The ability to accurately image rectus muscle insertions has significant implications for the management of strabismus patients. 

UBM, CT, MRI, and B-scan ultrasound can also be used to image the EOMs. These methods have advantages and disadvantages. The advantages of some include the ability to image the entire EOM, orbits, and pulley system; however, these techniques can be difficult to perform in certain patients due to claustrophobia, requirement for sedation or general anesthesia, and ionizing radiation. OCT can be obtained by a minimally invasive, quicker approach, and can be used pre-operatively and post-operatively in cooperative patients (3 years and older in this study), as well as intra-operatively while the patient is already under general anesthesia. 

However, the OCT had limitations—rectus muscles are not always able to be imaged, so the accuracy was limited (42–84% of insertions were ≤1 mm of the OCT measurement, depending on the machine). In some cases, the muscle insertion had an ambiguous transition from muscle tendon to sclera or Tenon’s capsule on the OCT image, or could not be identified, which was not useful. In contrast, in other cases the insertion was very distinct and provided more definitive information with higher level of confidence to the surgeon. The limitation of ambiguous, unidentified, or misidentified insertion may be improved with newer, higher-resolution OCT technology that continues to develop. The limitations of OCT also include the inability to image the oblique muscles or posterior portions of the EOMs. Also, many previously recessed muscles were not able to be imaged posteriorly enough to capture the new muscle insertion (only 17/32 rectus muscles ≥10 mm from the limbus could be imaged), with less success post-operatively than pre-operatively. However, the inability to image the rectus insertion a certain distance from the limbus can provide useful information for future surgical planning.

Our results of 61% (62/102) of imaged muscles being at ≤1 mm difference is not as successful as previous studies by Ngo et al., at 89.9% for pediatric patients, or Rosetto et al. at 77% and Pihlblad et al. at 70% on an adult populations [13,21,27]. Our results add to the literature on this subject and add previously unreported results using the Biotogen and Rescan OCT machines. 

The OCT machines each have their individual advantages and disadvantages, as well. The Spectralis and Visante can only be used in an outpatient setting, and the Rescan only in the operating room, but the Bioptogen can be used in both. Younger and uncooperative patients (seven patients under 3 years old in our study and one with developmental delay) were not able to be imaged as outpatients. Having the information before going to surgery is helpful for planning, but intraoperative information with the patient already under anesthesia for the planned surgery can also be helpful. The Rescan can be used while operating with real-time information, which can be very advantageous in some circumstances. The results also show that many different OCT machines that are available are capable of imaging rectus muscle insertions. Within the limitations discussed above, the ability to image the rectus muscle insertions pre-operatively, intra-operatively, and post-operatively in a non-invasive way gives valuable information to the strabismus surgeon to plan surgery, execute the surgery, and interpret the surgical outcomes. 

Some limitations of the study include that measurements were all performed by the same pediatric ophthalmologist, who was not blinded to the patient or surgery. The study had a small number of subjects, and an even smaller amount that were successfully imaged by all four machines (Table 2). The study also assumed the rectus muscle insertion did not change post-operatively. 

In conclusion, different OCT machines are capable of imaging rectus muscle insertions pre-operatively, intra-operatively, and post-operatively, which gives additional useful information to the strabismus surgeon. To our knowledge, this is the first study to use an operating microscope with integrated intra-operative OCT to image rectus muscle insertions. As technology continues to advance, we hope to be able to image muscle insertions with greater accuracy. Further studies will be needed to evaluate the different available and emerging techniques for imaging muscle insertions, in order to further refine our surgical approach. 

## 5. Conclusions

OCT machines are capable of imaging the rectus muscle insertions pre-operatively, intra-operatively, and post-operatively. The muscle insertion information provided by the different OCT machines can be advantageous to the strabismus surgeon, and may help with planning surgery, performing surgery and interpreting surgical outcomes. 

## Figures and Tables

**Table 1:** Anterior segment optical coherence tomography measurement results.

	Bioptigen	Rescan	Spectralis	Visante
Able to image muscle insertion	24/34 (71%)	25/28 (89%)	22/38 (58%)	31/40 (78%)
Mean difference between OCT measurement and caliper (SD)	0.06 mm (1.81) range −4.0 to +3.5	0.43 mm (1.27) range −2.5 to +3.5	0.47 mm (1.54) range −3.7 to +4.3	−0.56 mm (1.9) range −4.0 to +5.0
Intraclass correlation coefficient (ICC)	0.62	0.83	0.85	0.88
*p*-value	<0.01	<0.001	<0.001	<0.001
≤1 mm	13/24 (54%)	21/25 (84%)	15/22 (68%)	13/31 (42%)
≤2 mm	18/24 (75%)	21/25 (84%)	20/22 (91%)	22/31 (71%)
Pre-op or before surgery on unoperated muscles: ≤1 mm	10/29 (34%)	19/24 (79%)	8/9 (89%)	5/11 (45%)
Post-op: ≤1 mm	^a^	^a^	7/13 (54%)	5/16 (31%)
Muscles imaged with all four machines (% ≤ 1 mm)	37.5% (3/8)	75% (6/8)	62.5% (5/8)	37.5% (3/8)

^a^ Only had intra-operative measurements.

**Table 2:** Patients able to be imaged with all four optical coherence tomography machines.

Patients	Intra-Op Measurment	Bioptigen	Rescan	Spectralis	Visante
Patient 1: LR ^a^	12 mm	Unable	9.0 mm	Unable	9.5 mm
Patient 2: MR	4.5 mm	Unable	3.5 mm	5.2 mm	6.4 mm
Patient 2: LR	7.0 mm	10.0 mm	8.0 mm	7.3 mm	10.7 mm
Patient 3: MR ^a^	9.5 mm	10.5 mm	6.0 mm	Unable	9.1 mm
Patient 3: LR	6.0 mm	6.0 mm	5.0 mm	6.0 mm	6.1 mm
Patient 4: MR	5.0 mm	4.5 mm	4.5 mm	4.1 mm	6.6 mm
Patient 4: LR	6.0 mm	unable	6.0 mm	6.4 mm	7.9 mm
Patient 4: IR	5.0 mm	unable	4.0 mm	Unable	5.8 mm

^a^ previously operated muscle, LR: lateral rectus, MR: medial rectus, IR: inferior rectus.

## References

[1] Leffler C.T., Vaziri K., Schwartz S.G., Cavuoto K.M., McKeown C.A., Kishor K.S., Janot A.C. (2016). Rates of Reoperation and Abnormal Binocularity Following Strabismus Surgery in Children. Am. J. Ophthalmol..

[2] Demer J.L., Clark R.A., Kono R., Wright W., Velez F., Rosenbaum A.L. (2002). A 12-year, prospective study of extraocular muscle imaging in complex strabismus. J. Aapos Off. Publ. Am. Assoc. Pediatric Ophthalmol. Strabismus.

[3] Ettl A., Kramer J., Daxer A., Koornneef L. (1997). High-resolution magnetic resonance imaging of the normal extraocular musculature. Eye.

[4] Demer J.L., Ortube M.C., Engle E.C., Thacker N. (2006). High-resolution magnetic resonance imaging demonstrates abnormalities of motor nerves and extraocular muscles in patients with neuropathic strabismus. J. Aapos Off. Publ. Am. Assoc. Pediatric Ophthalmol. Strabismus.

[5] Demer J.L., Dushyanth A. (2011). T2-weighted fast spin-echo magnetic resonance imaging of extraocular muscles. J. Aapos Off. Publ. Am. Assoc. Pediatric Ophthalmol. Strabismus.

[6] Tamburrelli C., Salgarello T., Vaiano A.S., Scullica L., Palombi M., Bagolini B. (2003). Ultrasound of the horizontal rectus muscle insertion sites: Implications in preoperative assessment of strabismus. Investig. Ophthalmol. Vis. Sci..

[7] Sacca S., Polizzi A., Macri A., Patrone G., Rolando M. (2000). Echographic study of extraocular muscle thickness in children and adults. Eye.

[8] Khan H.A., Smith D.R., Kraft S.P. (2012). Localising rectus muscle insertions using high frequency wide-field ultrasound biomicroscopy. Br. J. Ophthalmol..

[9] Watts P., Smith D., Mackeen L., Kraft S., Buncic J.R., Abdolell M. (2002). Evaluation of the ultrasound biomicroscope in strabismus surgery. J. Aapos Off. Publ. Am. Assoc. Pediatric Ophthalmol. Strabismus.

[10] Dai S., Kraft S.P., Smith D.R., Buncic J.R. (2006). Ultrasound biomicroscopy in strabismus reoperations. J. Aapos Off. Publ. Am. Assoc. Pediatric Ophthalmol. Strabismus.

[11] Solarte C.E., Smith D.R., Buncic J.R., Tehrani N.N., Kraft S.P. (2008). Evaluation of vertical rectus muscles using ultrasound biomicroscopy. J. Aapos Off. Publ. Am. Assoc. Pediatric Ophthalmol. Strabismus.

[12] Takkar B., Sharma P., Singh A.K., Sahay P. (2016). Anterior segment optical coherence tomography for identifying muscle status in strabismus surgery. Int. J. Ophthalmol..

[13] Ngo C.S., Smith D., Kraft S.P. (2015). The accuracy of anterior segment optical coherence tomography (AS-OCT) in localizing extraocular rectus muscles insertions. J. Aapos Off. Publ. Am. Assoc. Pediatric Ophthalmol. Strabismus.

[14] Pihlblad M.S., Erenler F., Sharma A., Manchandia A., Reynolds J.D. (2016). Anterior Segment Optical Coherence Tomography of the Horizontal and Vertical Extraocular Muscles With Measurement of the Insertion to Limbus Distance. J. Pediatric Ophthalmol. Strabismus.

[15] Liu X., Wang F., Xiao Y., Ye X., Hou L. (2011). Measurement of the limbus-insertion distance in adult strabismus patients with anterior segment optical coherence tomography. Investig. Ophthalmol. Vis. Sci..

[16] Park K.A., Lee J.Y., Oh S.Y. (2014). Reproducibility of horizontal extraocular muscle insertion distance in anterior segment optical coherence tomography and the effect of head position. J. Aapos Off. Publ. Am. Assoc. Pediatric Ophthalmol. Strabismus.

[17] Salcedo-Villanueva G., Paciuc-Beja M., Harasawa M., Velez-Montoya R., Olson J.L., Oliver S.C., Mandava N., Quiroz-Mercado H. (2015). Identification and biometry of horizontal extraocular muscle tendons using optical coherence tomography. Graefe’s Arch. Clin. Exp. Ophthalmol..

[18] Haner N.U., Dysli M., Abegg M., Zinkernagel M.S. (2015). Enhanced-depth optical coherence tomography for imaging horizontal rectus muscles in Graves’ orbitopathy. Graefe’s Arch. Clin. Exp. Ophthalmol..

[19] Radhakrishnan S., Rollins A.M., Roth J.E., Yazdanfar S., Westphal V., Bardenstein D.S., Izatt J.A. (2001). Real-time optical coherence tomography of the anterior segment at 1310 nm. Arch. Ophthalmol..

[20] De-Pablo-Gomez-de-Liano L., Fernandez-Vigo J.I., Ventura-Abreu N., Morales-Fernandez L., Fernandez-Perez C., Garcia-Feijoo J., Gomez-de-Liano R. (2016). Spectral domain optical coherence tomography to assess the insertion of extraocular rectus muscles. J. Aapos Off. Publ. Am. Assoc. Pediatric Ophthalmol. Strabismus.

[21] Pihlblad M.S., Reynolds J.D. (2017). Anterior Segment Optical Coherence Tomography of Previously Operated Extraocular Muscles. Am. Orthopt. J..

[22] Ocak O.B., Inal A., Yilmaz I., Aygit E.D., Ocak S.Y., Celik S., Taskapili M., Gokyigit B. (2019). Measurement of extraocular horizontal muscle insertion distance via anterior segment optical coherence tomography of healthy children and comparison with healthy adults. Int. Ophthalmol..

[23] De-Pablo-Gomez-de-Liano L., Fernandez-Vigo J.I., Ventura-Abreu N., Troyano-Rivas J., Nino-Rueda C., Romo-Lopez A., Gomez-de-Liano R. (2018). Optical Coherence Tomography Thickness Measurements of the Extraocular Rectus Muscle Tendons in Graves’ Ophthalmopathy. J. Pediatric Ophthalmol. Strabismus.

[24] Han J.Y., Lee D.C., Lee S.Y. (2018). Horizontal Extraocular Muscle and Scleral Anatomy in Children: A Swept-Source Anterior Segment Optical Coherence Tomography Study. Korean J. Ophthalmol..

[25] Venincasa M.J., Osigian C.J., Cavuoto K.M., Rossetto J.D., Capo H. (2017). Combination of anterior segment optical coherence tomography modalities to improve accuracy of rectus muscle insertion location. J. Aapos Off. Publ. Am. Assoc. Pediatric Ophthalmol. Strabismus.

[26] Lee J.Y., Park K.A., Lyu I.J., Oh S.Y. (2017). Postoperative change in lateral rectus muscle insertion measured by anterior segment optical coherence tomography. Eye.

[27] Rossetto J.D., Cavuoto K.M., Allemann N., McKeown C.A., Capo H. (2017). Accuracy of Optical Coherence Tomography Measurements of Rectus Muscle Insertions in Adult Patients Undergoing Strabismus Surgery. Am. J. Ophthalmol..

[28] De-Pablo-Gomez-de-Liano L., Fernandez-Vigo J.I., Ventura-Abreu N., Garcia-Feijoo J., Fernandez-Vigo J.A., Gomez-de-Liano R. (2017). Agreement Between Three Optical Coherence Tomography Devices to Assess the Insertion Distance and Thickness of Horizontal Rectus Muscles. J. Pediatric Ophthalmol. Strabismus.

[29] De-Pablo-Gomez-de-Liano L., Fernandez-Vigo J.I., Ventura-Abreu N., Morales-Fernandez L., Garcia-Feijoo J., Gomez-de-Liano R. (2016). Agreement between intraoperative measurements and optical coherence tomography of the limbus-insertion distance of the extraocular muscles. Arch. De La Soc. Esp. De Oftalmol..

